# Differential effects of vagus nerve stimulation strategies on glycemia and pancreatic secretions

**DOI:** 10.14814/phy2.14479

**Published:** 2020-06-08

**Authors:** Sophie C. Payne, Glenn Ward, Richard J. MacIsaac, Tomoko Hyakumura, James B. Fallon, Joel Villalobos

**Affiliations:** ^1^ Bionics Institute East Melbourne Vic. Australia; ^2^ Medical Bionics Department The University of Melbourne Parkville Vic. Australia; ^3^ Department of Endocrinology and Diabetes St Vincent’s Hospital Fitzroy Vic. Australia; ^4^ Department of Medicine The University of Melbourne Parkville Vic. Australia

**Keywords:** bioelectronic medicine, medical devices, peripheral nerve stimulation, type 2 diabetes mellitus

## Abstract

Despite advancements in pharmacotherapies, glycemia is poorly controlled in type 2 diabetic patients. As the vagus nerve regulates energy metabolism, here we evaluated the effect various electrical vagus nerve stimulation strategies have on glycemia and glucose‐regulating hormones, as a first step to developing a novel therapy of type 2 diabetes. Sprague–Dawley rats were anesthetized, the abdominal (anterior) vagus nerve implanted, and various stimulation strategies applied to the nerve: (a) 15 Hz; (b) 4 kHz, or 40 kHz and; (c) a combination of 15 Hz and 40 kHz to directionally activate afferent or efferent vagal fibers. Following a glucose bolus (500 mg/kg, I.V.), stimulation strategies were applied (60 min) and serial blood samples taken. No stimulation was used as a crossover control sequence. Applying 15 Hz stimulation significantly increased glucose (+2.9 ± 0.2 mM·hr, *p* = .015) and glucagon (+17.1 ± 8.0 pg·hr/ml, *p* = .022), compared to no stimulation. Application of 4 kHz stimulation also significantly increased glucose levels (+1.5 ± 0.5 mM·hr, *p* = .049), while 40 kHz frequency stimulation resulted in no changes to glucose levels but did significantly lower glucagon (−12.3 ± 1.1 pg·hr/ml, *p* = .0009). Directional afferent stimulation increased glucose (+2.4 ± 1.5 mM·hr) and glucagon levels (+39.5 ± 15.0 pg·hr/ml). Despite hyperglycemia resulting when VNS, aVNS, and 4 kHz stimulation strategies were applied, the changes in insulin levels were not significant (*p* ≥ .05). In summary, vagus nerve stimulation modulates glycemia by effecting glucagon and insulin secretions, and high‐frequency 40 kHz stimulation may have potential application for the treatment of type 2 diabetes.

## Graphical abstract

Blood glucose can be controlled by electrically stimulating the vagus nerve. This approach could be a promising therapy of type 2 diabetes.

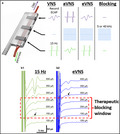

## 1 INTRODUCTION

Diabetes mellitus is a chronic, progressive condition that affects over 350 million people worldwide. It is one of the most prevalent chronic diseases, with around 1 million Australians currently diagnosed (Zheng, Ley, & Hu, 2018). The majority (90%) of diabetic patients suffer from the type 2 form of the disease, which is associated with poor diet, ageing, and genetic predisposition (Zheng et al., 2018). During early stages of type 2 diabetes, insulin‐mediated glucose uptake in the liver, muscle, and adipose tissue becomes less effective, a state named insulin resistance. The high levels of insulin secreted in compensation lead to subsequent dysfunction of β‐cells in the pancreas, which eventually leads to a permanent reduction in insulin secretion (Stumvoll, Goldstein, & van Haeften, 2005). The resulting high glycemic levels damage vital organs over time and can lead to serious complications such as the development of cardiovascular disease, retinopathy, and neuropathy (Zheng et al., 2018). A strategy for many therapies of type 2 diabetes is to modulate endogenous hormonal secretions from the pancreas to improve glycemic control in type 2 diabetes (Vetere, Choudhary, Burns, & Wagner, 2014).

Despite advancements in pharmacological therapies, up to half of type 2 diabetic patients fail to achieve adequate glycemic control due to poor medication adherence (Polonsky & Henry, 2016) or secondary failure of pharmacological treatments (Brown, Conner, & Nichols, 2010; Harrower, 1994). Medical adherence is worsened by the experience of unpleasant side effects, such as hypoglycemia (Polonsky & Henry, 2016; Walz et al., 2014), and with greater complexity of the medication administration regimen (Claxton, Cramer, & Pierce, 2001; Coleman et al., 2012; de Vries et al., 2014; Garcia‐Perez, Alvarez, Dilla, Gil‐Guillen, & Orozco‐Beltran, 2013). Clinical management of type 2 diabetes usually involves a combination of drugs to improve glycemic control and each has its associated side effects. Antihyperglycemic drugs (biguanides) are associated with gastrointestinal dysfunctions such as diarrhea, nausea, and vomiting (Siavash, Tabbakhian, Sabzghabaee, & Razavi, 2017). Hypoglycemic drugs (sulfonylureas) are strongly associated with uncontrolled hypoglycemic episodes, which can lead to loss of consciousness, coma, and even death (Harrigan, Nathan, & Beattie, 2001; Spiller & Sawyer, 2006). This is also an issue with antihyperglycemic medication, where nearly a third of patients experience hypoglycemic side effects (Chao, Nau, & Aikens, 2007). Severely unpleasant side effects can lead to unreliable administration (i.e., skipping of doses) or even discontinuation of the therapy (Siavash et al., 2017). It is clear that a new therapy is needed to improve medical adherence, reduce secondary failure, and minimize side effects while avoiding hypoglycemic episodes. These are unmet clinical needs that cause suboptimal glycemic control and contribute to worsening of the disease.

Electrical stimulation of the vagus nerve has regulatory approval for the treatment of epilepsy, depression, and obesity (Payne, Furness, & Stebbing, 2019). However, the vagus nerve is also implicated in the regulation of energy metabolism, food intake, and glycemia as the nerve has a major role in the control of pancreatic hormonal secretions (Waise, Dranse, & Lam, 2018). Early studies in rats and dogs show that electrical stimulation of the vagus nerve effects the secretion of insulin and glucagon from the pancreas (Ahren, Paquette, & Taborsky, 1986; Ionescu, Rohner‐Jeanrenaud, Berthoud, & Jeanrenaud, 1983; Nishi et al., 1987; Rozman, Bunc, & Zorko, 2004). However, the results of previous studies examining how vagus nerve stimulation regulates glycemia and glucose metabolism hormones have revealed conflicting results (Table 1). Vagus nerve stimulation delivered at low frequencies (5–30 Hz) activates both afferent fibers that send signals toward the brain, and efferent fibers that send signals toward the pancreas and other vital organs (Bonaz, Picq, Sinniger, Mayol, & Clarencon, 2013). Cervical vagus nerve stimulation at 5 Hz elevated fasting blood glucose in rats (Stauss, Stangl, Clark, Kwitek, & Lira, 2018). However, 30 Hz frequency abdominal stimulation reduced fasted blood glucose in obese mini‐pigs (Malbert, Picq, Divoux, Henry, & Horowitz, 2017) and 5 Hz stimulation decreased glycemic response to an oral glucose tolerance test type 2 diabetic rats (Yin, Ji, Gharibani, & Chen, 2019). An important distinction in the role of afferent and efferent vagal signaling was evidenced by stimulating only the proximal or distal cut end of the cervical rat vagus nerve at 5 Hz, which resulted in an afferent‐driven increase in glycemia or an efferent‐driven decrease in glycemia due to corresponding changes in pancreatic secretions (Meyers, Kronemberger, Lira, Rahmouni, & Stauss, 2016). In a different approach, high‐frequency stimulation in the kilohertz range is presumed to block nerve activity (Camilleri et al., 2008; Tweden, Anvari, et al., 2006; Tweden, Sarr, et al., 2006). Stimulation at 5 kHz is thought to block afferent vagal ‘hunger’ signaling to the brain and is used clinically for the treatment of obesity (Camilleri et al., 2008; Payne, Furness, & Stebbing, 2019), but also reduces fasted glucose levels and glycated hemoglobin in diabetic patients (Shikora et al., 2013). As such, a better understanding of the effect of vagus nerve stimulation on glycemic levels and underlying hormone secretions is an important first step in the development of new treatments of the regulation of glycemia in type 2 diabetes.

**TABLE 1:** Effects of vagus nerve stimulation on glycemia and pancreatic hormone secretions

Reference	Species/model	Stim parameters	Stim site	Glucose	Insulin	Glucagon	GLP‐1
Ionescu et al. (1983)	Rat/normal	30 Hz, 50 µA, 0.2 ms	Dorsal motor nucleus	**↑**	**↑**	‐	‐
Ahren et al. (1986)	Dog/normal	10 Hz, 13.5 mA, 5 ms	Anterior and posterior thoracic vagus nerves	**↑**	**↑**	**↑**	‐
Nishi et al. (1987)	Rat/normal	10 Hz, 10 V, 1 ms	Subdiaphragmatic anterior and posterior vagus nerves	‐	**↑**	**↑**	‐
Rozman et al. (2004)	Dog/normal Alloxan	20 Hz, 1 mA, 200 µs	Cervical vagus nerve	**‐**	**↑ ↑**	**↑ ↑**	‐
Meyers et al. (2016)	Rat/normal	5 Hz, 3 V, 1 ms VNS aVNS^a^ eVNS^a^	Right or left cervical vagus nerve	**↑ ↑ =**	**= = ↑**	**↑ = ↑**	‐
Stauss et al. (2018)	Rats/normal	5 Hz, 3V, 1 ms	Right cervical vagus nerve	**↑**	**↓**	=	‐
This study	Rat/normal	15 Hz VNS aVNS eVNS 40 kHz 4 kHz	Subdiaphragmatic anterior vagus nerve	**↑ ↑ = = ↑**	**= = = = =**	**↑ ↑ = ↓ =**	**= = = = =**
Yin et al. (2019)	Rats/normal T2DM	5 Hz, 2 mA, 0.3 ms	Subdiaphragmatic anterior vagus nerve	**↓ ↓**	‐ **↓**	‐	
Malbert et al. (2017)	Mini pigs/obese	30 Hz	Subdiaphragmatic anterior and posterior vagus nerves	**↓**	**↓**	‐	‐
Shikora et al. (2013)	Human/obese & T2DM	5 kHz, 3–8 mA	Anterior and posterior gastric vagus nerves	**↓**	‐	‐	‐

^a^ Experiment stimulated the cut proximal and distal ends of the cervical vagus nerve. Arrows (**↑ ↓**) indicate a significant increase or decrease (*p* > .05), an equal sign (=) has no statistically significant change (*p* ≥ .05), and a dash (‐) indicates data were not assessed.

To date, there is no one comparative study that assesses the effects of various electrical stimulation strategies of the abdominal vagus nerve on glycemia and pancreatic secretions (Table 1). Furthermore, a notable absence in these previous studies is a report of how the incretin hormone GLP‐1 is affected by stimulation (Table 1). Incretin hormones are released from the small intestine to trigger the additional release of insulin during postprandial phases (Berthoud, 2008) and can be effected by electrical stimulation of the vagus nerve (Rocca & Brubaker, 1999). As such, here we will examine changes in the levels of glucose, insulin, glucagon, and GLP‐1 during the application of: (a) low‐frequency 15 Hz stimulation (‘VNS’), designed to increase activity in the vagus nerve; (b) a combination of low (15 Hz)‐ and high (40 kHz)‐frequency stimulation, designed to provide ‘directional’ activation in which afferent (‘aVNS’) or efferent (‘eVNS’) fibers are primarily activated (Patel, Saxena, Bellamkonda, & Butera, 2017); (c) high‐frequency 40 kHz stimulation, in which kilohertz frequency alternating current is applied to produce a focal block on the nerve and inhibit activity (Kilgore & Bhadra, 2014), and high‐frequency ‘4 kHz stimulation, which mimicked the commercially available “vBloc maestro” stimulation strategy for the treatment of obesity’ (Camilleri et al., 2008; Ikramuddin et al., 2014). As such, this study seeks to determine the effects of various vagus nerve stimulation strategies for modulating glycemia and provide a comparative study with the aim to establish a basis for clinical translation.

## 2 MATERIALS AND METHODS

### 2.1 Animals and anesthesia

All experiments used male Sprague–Dawley rats (8–10 weeks, Animal Resource Centre, Western Australia). Procedures were approved by the Bionics Institute Animal Research Ethics Committee (17‐369AB, 18‐384AB) and complied with the Australian Code for the Care and Use of Animals for Scientific Purposes (National Health and Medical Research Council of Australia) and the Prevention of Cruelty to Animals (1986) Act. Animals were kept on a 12 hr light/dark cycle and allowed access to standard chow and water ad libitum. Rats were fasted overnight (14–16 hr) and anesthetized (1.5%–2% isoflurane in oxygen flowing at 1 L/min) prior to the terminal surgical procedure. Breathing rate was maintained between 45 and 60 breaths per minute for the duration of the nonrecovery experiment. At the conclusion of the experiment, rats were euthanized (300 mg/kg Lethabarb, intravenous injection).

### 2.2 Vagus nerve electrode array

**FIGURE 1:**
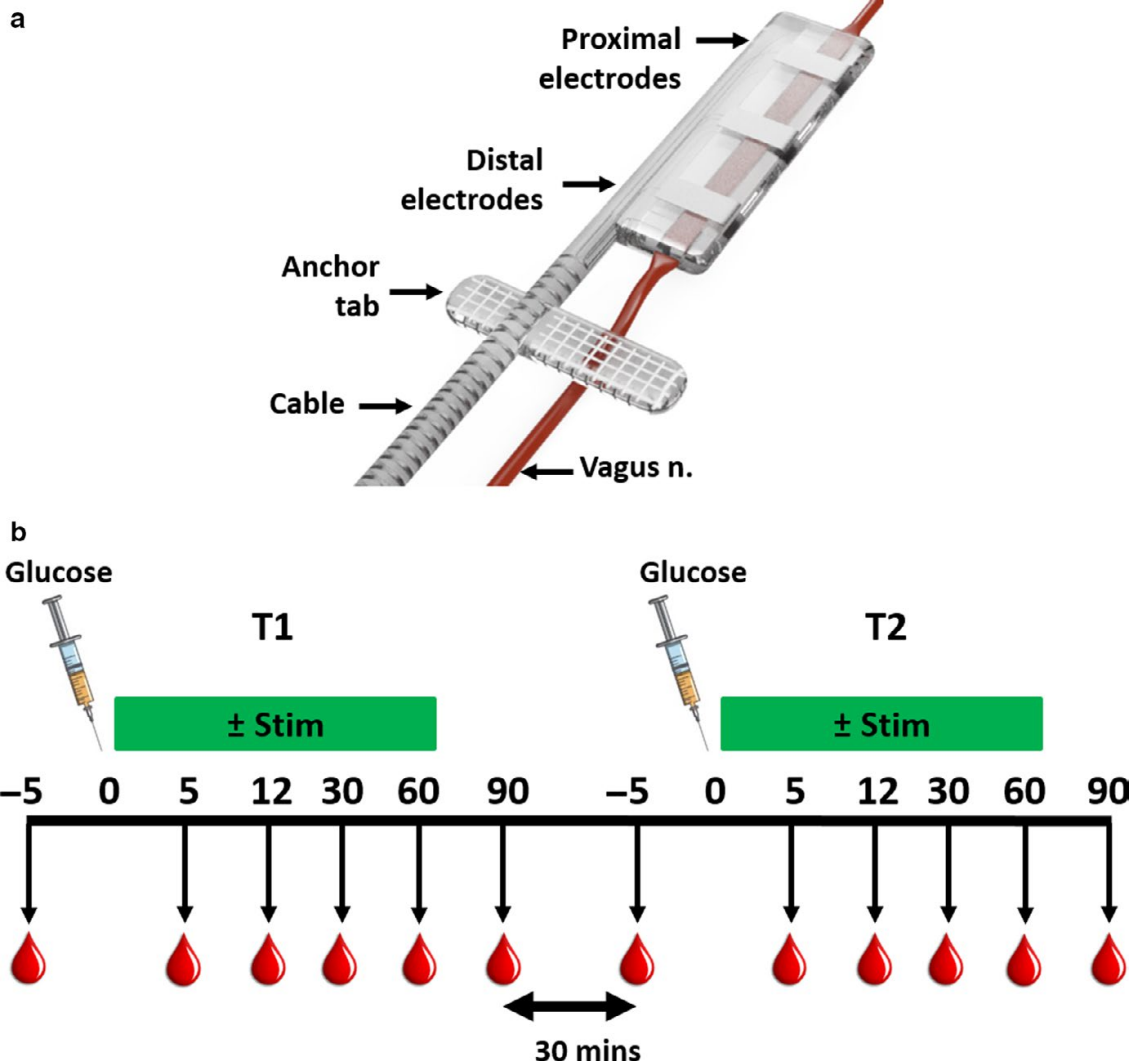
Vagus nerve array and experimental design. (a) The cuff electrode array had six platinum electrodes arranged in pairs. The anchor tab was sutured to the esophagus to provide mechanical support. (b) The crossover control sequence experimental design shows stimulation was applied continuously for 60 min immediately following intravenous glucose bolus (indicated by the syringe icon). In test 2 (T2), the intervention applied was interchanged with no‐stimulation control

The vagus nerve electrode array consisted of six platinum (99.95%) electrodes embedded into a medical‐grade silicone‐elastomer cuff. Each platinum electrode had an exposed surface area of 0.39 mm^2^. The electrodes were arranged in pairs, on opposite sides of the cuff, and with a distance of 3.4 mm between electrode pairs (Figure 1a). A rectangular‐section lumen (0.55 mm × 0.2 mm) traversed the cuff for housing the vagus nerve. The silicone cuff was sutured closed to prevent the nerve from migrating out of the cuff. A silicone suturing tab on the lead was used to anchor the array to the esophagus to provide mechanical stability. A helical cable with 25‐µm‐diameter platinum/iridium (90/10) wires from the electrodes was tunneled to a percutaneous connector mounted on the lumbar region of the rat.

### 2.3 Surgical interventions

In brief, the ventral abdominal midline was incised and the ventral esophagus and subdiaphragmatic anterior abdominal branch of the vagus nerve exposed, similar to that done previously (Payne, Furness, Burns, et al., 2019). The nerve was dissected away from the esophagus and the array implanted rostral to the hepatic and celiac branches of the vagus. The array was sutured (7‐0 silk, Ethicon) to the esophagus to provide stabilization and the abdominal cavity and skin sutured closed. As a method to withdraw venous blood and deliver the glucose bolus, the left and right femoral veins were exposed and cannulated. The cannula lines were kept patent during the experiment by using sterile saline and heparin (10 units/ml). Animals were kept hydrated and blood loss replaced with similar volumes of sterile Hartman's solution. Surgical implantation of the electrode cuff on the abdominal vagus was successful in all the 33 naïve animals used. Two animals were excluded from the experimental dataset due to anesthetic issues and one due to a diabetic‐type response during the initial glucose tolerance test (blood glucose > 30 mM sustained more than 2 hr after an intravenous (i.v.) bolus).

### 2.4 Glucose tolerance test

At *T* = −5 min, control baseline blood samples were taken. An intravenous bolus of glucose (500 mg/kg) was administered at *T* = 0, and a series of blood samples were taken at *T* = 5, 12, 30, 60, and 90 min (Figure 1b). Vagus nerve stimulation (details provided below) was applied for 60 min from the glucose bolus. A sequential glucose bolus was given at least 90 min apart, where one post bolus period (Test 1, T1) included electrical stimulation and the other acted as an unstimulated control (Test 2, T2). The order alternated between experiments to act as cross‐over control sequence (Figure 1b).

### 2.5 Electrode impedance testing and electrophysiological recordings

Functionality of electrodes was tested by measuring the impedance from voltage transients to current pulses (25 µs, 100 µA), in common‐ground configuration (one active electrode vs. all others as return) as was reported previously (Fallon, Irvine, & Shepherd, 2009). Electrically evoked compound action potentials (ECAPs) were recorded to determine threshold of neural activation. ECAPs were generated by stimulating the proximal electrode pair (200 µs; 15 Hz; 0–2 mA) and recording from the distal pair (averaged over 100 pulses and repeated twice), closest to the pancreas. Recordings were sampled at a rate of 200 kHz and digitally filtered (20–2,000 Hz band pass; Payne, Furness, Burns, et al., 2019). The ECAP threshold was defined as the minimum stimulus intensity producing a response amplitude of at least 2 μV within a poststimulus latency window of 4–10 ms. This latency corresponds to conduction velocities within the range of C‐fiber responses (Castoro et al., 2011). In all experiments the stimulation current was suprathreshold (detailed in the next section).

### 2.6 Vagus nerve stimulation strategies

**FIGURE 2:**
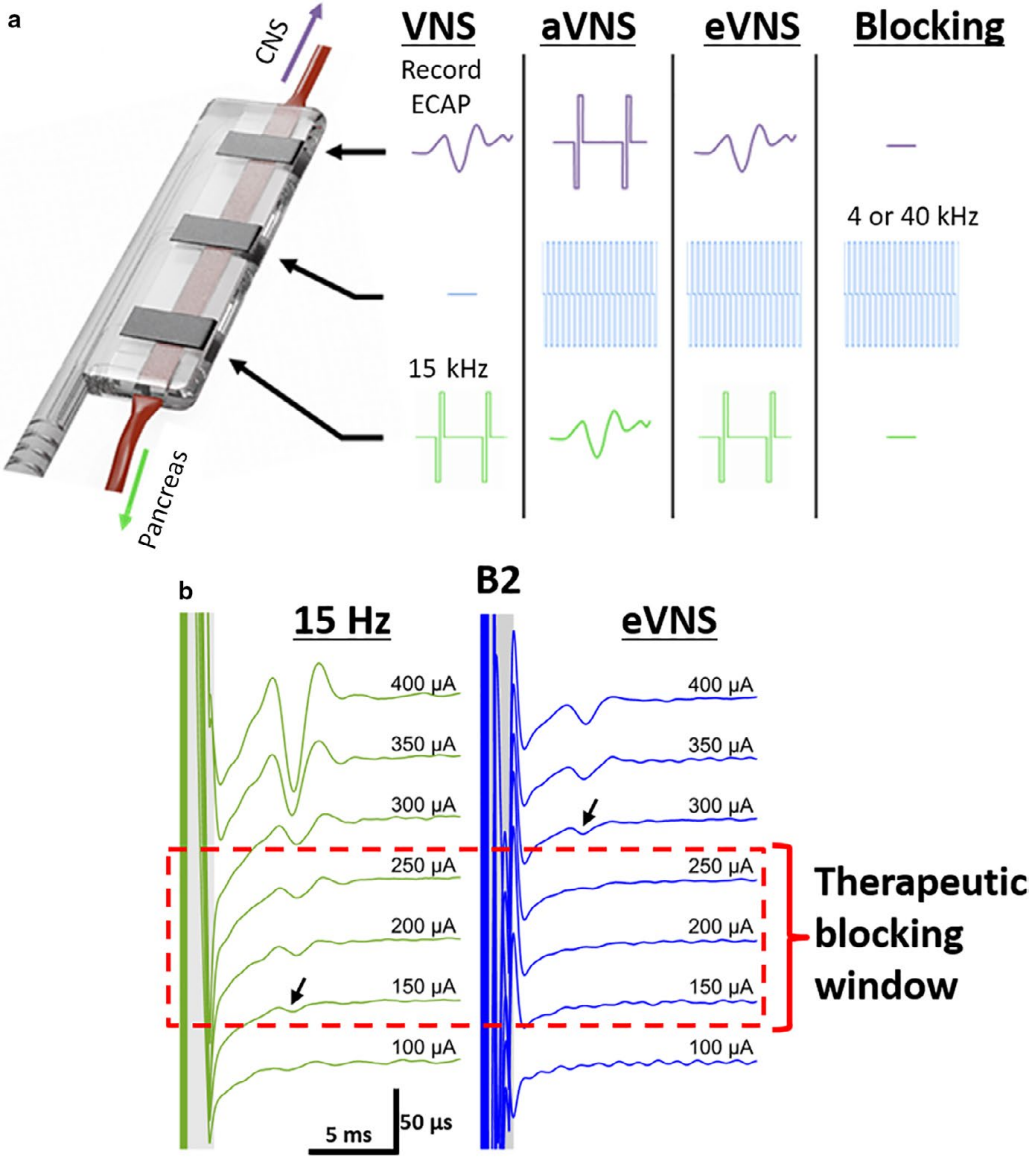
Electrical stimulation strategies applied to the abdominal vagus nerve. (a) Schematic diagram indicates the stimulation strategies that were applied to the abdominal vagus nerve to effect change in glucose and pancreatic output. Directional stimulation of vagal fibres was achieved by applying 40 kHz to the middle electrode pair and 15 Hz stimulation to another electrode pair. (b1,b2) Traces show an example from one rat. The ‘therapeutic blocking window’ is the range of current levels that can be applied to allow for directionally activating the nerve and is defined as the range between neural activation threshold (arrow) generated during no blocking (b1: 150 μA) and neural threshold when combined with 40 kHz stimulation (b2, 300 μA) i.e. above 150 μA but below 300 μA

A custom‐made external stimulator was used to deliver biphasic current pulses at minimum 15 Hz and up to 4 kHz (Fallon et al., 2018). For even higher‐frequency stimulation, a 40 kHz sinusoid waveform was output from a function generator and isolated via a transformer. The following stimulation strategies were used: (a) Low‐frequency stimulation, referred to as ‘VNS’, applied at 15 Hz, 200 µs biphasic square pulses at 1 mA (Figure 2a); (b) High‐frequency 40 kHz stimulation, referred to as ‘40 kHz’, applied at 40 kHz sinusoidal frequency and a current of 4 mA (peak to peak; Figure 2a); (c) High‐frequency 4 kHz stimulation, referred to as ‘4 kHz’, applied 4 kHz of 100 µs, 2 mA, biphasic square pulses (Figure 2a); (d) Directional afferent stimulation, referred to as ‘aVNS’, simultaneously applied 40 kHz sinusoidal current of 4 mA (peak to peak) to the central electrode pair and 15 Hz, 200 µs square biphasic pulses to the proximal electrode pair (Figures 1a and 2a). Current level applied was within the ‘therapeutic blocking window’ (see below for details) and; (e) directional efferent stimulation, referred to as ‘eVNS’, simultaneously applied 40 kHz sinusoidal current of 4 mA (peak to peak) to the central electrode pair and 15 Hz, 200 µs biphasic square pulses to the distal electrode pair (Figures 1a and 2a,b1,b2). Current level applied was within the ‘therapeutic blocking window’ (see next paragraph for details; Figure 2b1,b2).

### 2.7 Therapeutic blocking window

The therapeutic blocking window was determined by assessing the neural response threshold when blocking was applied (Figure 2b2), compared to when no blocking is applied (Figure 2b1). Application of 40 kHz stimulation to the nerve was only effective in blocking the nerve activity induced by 15 Hz stimulation below a certain current level. The ‘therapeutic blocking window’ refers to the range of current levels that elicited nerve activity at 15 Hz, but where the activity was not observed when combined with 40 kHz stimulation, to remain efficient at blocking the nerve in one direction. The example in Figure 2b shows the neural threshold is 150 µA (Figure 2b1) during no blocking, but increased to 300 µA when 40 kHz stimulation was used to block afferent activity (Figure 2b2). Therefore, in this example applying 15 Hz and a current level higher than 150 µA but lower than 300 µA (i.e., the therapeutic blocking window) will be effective in directionally activating the nerve. The therapeutic blocking threshold is determined separately in each subject, as the threshold will differ between rats.

### 2.8 Quantification of glycemia and hormones

Whole blood samples (300 µl) from the femoral vein were collected in K2‐EDTA coated tubes (Starstedt). Glycemia was measured using a glucometer (Accu‐Chek Nano, Roche), and the remaining blood was centrifuged (2,000 *g* for 10 min), plasma aliquoted, and stored at −80°C. To validate the use of the glucometer used (Accu‐Chek Nano, Roche), a subset of plasma samples was compared with a glucose assay (kit 81693, Crystal Chem) and were found to be highly correlated (Pearson *R^2^* = .85). On the day of the assay, aliquots were thawed and enzyme‐linked immunosorbent assays (ELISA) were performed according to manufacturer's instructions (Crystal Chem Inc) for insulin (kit: 90010), glucagon (kit: 81519), and GLP‐1 (kit: 81507). The hormone levels were determined via absorbance measurements using a FLUOStar Omega kinetic plate reader (BMG Labtech). For each glucose or hormone response, the baseline at *T* = −5 min was subtracted and the area under the curve calculated over the 60‐min period during which stimulation was applied.

### 2.9 Statistical analysis

The net effect of vagus nerve stimulation on the levels of glucose, glucagon, insulin, and GLP‐1 over the 60‐min testing period was compared to unstimulated response within the same rat using paired student *t* tests to assess differences. Statistically significant differences were accepted as *p* < .05 and GraphPad was Prism 4 (GraphPad Software) used for all analysis. Values are reported as mean ± standard error of the mean.

## 3 RESULTS

### 3.1 Impedance of implanted electrodes and electrically evoked neural responses

The impedance of electrodes in vitro (saline) was 4.5 ± 0.4 kΩ, while the impedance of the electrodes in vivo was 6.3 ± 2 kΩ. ECAPs were recorded to ensure stimulation levels remained above threshold. The mean threshold for evoking ECAP responses was 692 ± 406 µA. There were no changes in neural thresholds following the 60‐min application of 4 kHz or 40 kHz stimulation.

### 3.2 Effects of low‐frequency vagus nerve stimulation

**FIGURE 3:**
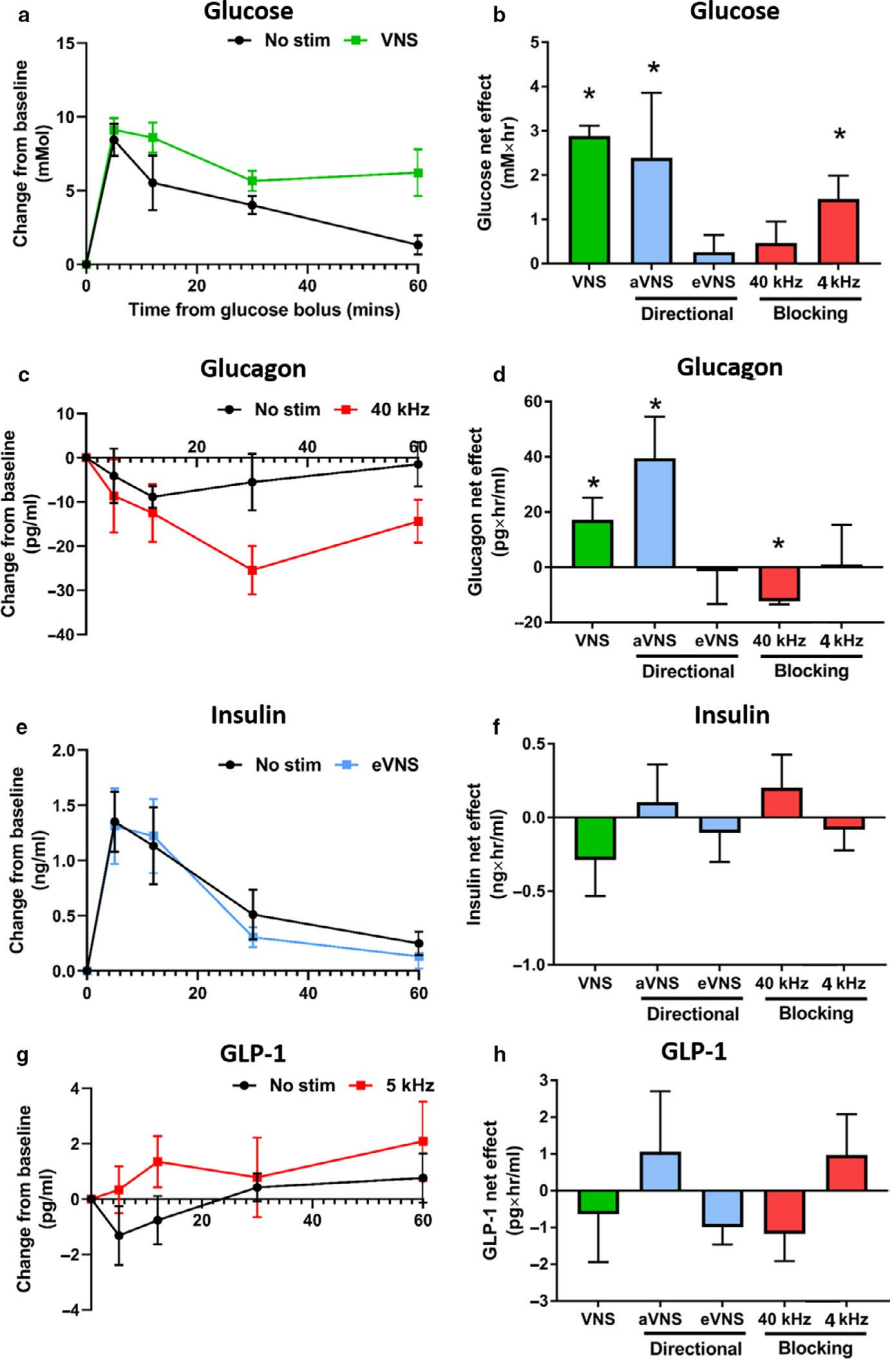
Changes in glucose and hormone levels during the application of 60 min of different vagus nerve stimulation strategies. (a,c,e,g) Selected graphs show examples of the stimulation strategy that had the largest effect on: glucose (a), glucagon (c), insulin (e) and GLP‐1 (g) during stimulation (indicated by coloured line) and no stimulation (indicated by black line), following an intravenous bolus of glucose. (b) Significant increases in glucose levels were detected during VNS (*p* = .015), aVNS (*p* = .01) and 4 kHz stimulation (*p* = .049). (d) Glucagon levels increased during aVNS (*p* = .043) but decreased during 40 kHz stimulation (*p* = .009). (f,h) There were no significant changes in insulin (*p* > .05, f) or GLP‐1 levels (*p* > .05, h) during the application of any stimulation strategies. Data in left graphs (a,c,e,g) show mean difference from baseline (T = 0) ± standard error of mean. Data in right graphs (b,d,f,h) show the delta difference in the response between unstimulated and stimulated tests over 60 min. Statistically significant differences (*p* < .05) were indicated by “*”

Low‐frequency 15 Hz stimulation produced a significant increase in glucose levels of +2.9 ± 0.23 mM·hr (+47%, paired *t* test *p* = .015; *n* = 5), a significant increase in glucagon of +17.1 ± 8.05 pg·hr/ml (+1,454%, *p* = .022), and no statistically significant changes in insulin (−0.29 ± 0.24 ng·hr/ml; −75%; *p* = .11) and GLP‐1 levels (−0.4 ± 1.31 pg·hr/ml; −342%; *p* = .12; Figure 3b,d,f,g).

### 3.3 Effects of high‐frequency vagus nerve stimulation

Application of 4 kHz and 40 kHz stimulation strategies had different effects on glucose and glucagon levels. Stimulation at 4 kHz (*n* = 5) caused a significant increase in glucose levels (+1.5 ± 0.5 mM·hr; +26% *p* = .049) that was accompanied by non‐statistically‐significant changes in glucagon (+1.1 ± 14.3 pg·hr/ml; +19%; *p* = .47), insulin (−0.08 ± 0.14 ng·hr/ml; −9%; *p* = .463), and GLP‐1 levels (+1.0 ± 1.1 pg·hr/ml; +153%; *p* = .251, Figure 3b,d,f,h). However, application of 40 kHz (*n* = 3) resulted in a statistically significant decrease in glucagon levels (−12.3 ± 1.2 pg·hr/ml; −249%; *p* = .009, Figure 3c,d). There were no statistically significant changes in glucose (+0.5 ± 0.49 mM·hr; +13%; *p* = .445), insulin (+0.20 ± 0.22 ng·hr/ml; +33%; *p* = .463 Figure 3e,f), and GLP‐1 levels (−1.20 ± 0.74 pg·hr/ml; −262%; *p* = .25) during 40 kHz stimulation.

### 3.4 Effects of directional nerve stimulation

A contrast was seen between the effects of afferent VNS (*n* = 7) and efferent VNS (*n* = 9) stimulation strategies on glucose and hormone levels. Application of aVNS produced a large and statistically significant increase in glucose levels of +2.4 ± 1.5 mM·hr (+63%; *p* < .01), which was accompanied by a significant increase in glucagon levels (+39 ± 15 pg·hr/ml; +485%; *p* = .04). There were no statistically significant changes in insulin (+0.10 ± 0.26 ng·hr/ml; +17%; *p* = .31) and GLP‐1 secretions (+1.1 ± 1.64 pg·hr/ml; 332%; *p* = .282; Figure 3b,d,f,h). In contrast, eVNS produced no statistically significant changes in glucose (+0.26 ± 0.38 mM·hr; 6%; *p* = .80, Figure 3a,b), glucagon (−1.4 ± 11.9 pg·hr/ml; −10%; *p* = .944), insulin (−0.10 ± 0.2 ng·hr/ml; −13%; *p* = .31), and GLP‐1 levels (−1.0 ± 0.5 pg·hr/ml; −186%; *p* = .130, Figure 3b,d,f,h).

## 4 DISCUSSION

The vagus nerve regulates energy metabolism, food intake, and glycemic control. Here, we found that applying different electrical stimulation strategies to the abdominal (anterior) vagus nerve modulates glycemia by effecting glucagon and insulin secretions. In particular, applying high‐frequency 40 kHz stimulation lowered glucagon secretions and may have a potential application for developing new treatments of type 2 diabetes.

The anterior abdominal vagus nerve resides below vagal branches to heart and lungs and avoids unwanted off‐target activation, and allows for the application of high levels of stimulation that may not be tolerated when applied to the cervical vagus nerve (Payne et al., 2018; Payne, Furness, Burns, et al., 2019; Payne, Furness, & Stebbing, 2019). Stimulation of the anterior subdiaphragmatic (abdominal) vagus nerve has previously been shown to decrease the glycemic response to an oral glucose tolerance test in type 2 diabetic rats (Yin et al., 2019). In rats, the majority of fibers from the left cervical vagus nerve form the anterior abdominal vagus, which branches into hepatic and celiac (and gastric) nerves (Berthoud & Neuhuber, 2000). The hepatic nerve innervates the liver, which has a major role in glucose homeostasis, and the head of the pancreas and the proximal duodenum (containing GLP‐producing intestinal cells; Berthoud & Neuhuber, 2000; Phillips, Baronowsky, & Powley, 1997), while the distal pancreatic lobe receives sparse innervation from the celiac nerve (Berthoud, Carlson, & Powley, 1991; Bockman, 1993; Teff and Teff, 2008). Although there are differences in the parasympathetic neural innervation of the human pancreas, electrical stimulation of cholinergic fibers significantly increased insulin sections in an isolated human pancreas model (Brunicardi, 1995).

Low‐frequency 15 Hz vagus nerve stimulation likely activated both afferent and efferent fibers, and caused a substantial increase in glycemia compared to unstimulated animals during a glucose challenge test. The hyperglycemic effect can be attributed to the activation of afferent vagal fibers, as a similar hyperglycemic response was observed during directional afferent, but not efferent, stimulation. Hepatic vagal afferents reportedly act as a glucose sensor, as the mean rate of hepatic neural activity decreased following an injection of glucagon to induce hyperglycemia, but increased in response to insulin‐induced hypoglycemia (Niijima, 1982, 1983). As such, electrically driven increases in vagal afferent activity may simulate a hypoglycemic state that leads to a feedback controlled increase in glycemia. Surprisingly, high‐frequency stimulation at 4 kHz, which is presumed to block the firing of afferent and efferent vagal fibers in humans (Camilleri et al., 2008, 2009), also resulted in hyperglycemia. However, here we suggest that applying 4 kHz was ineffective at blocking neural activity, and in instead promoted an increase in afferent fiber activity, which lead to the hyperglycemic effect observed in our experiments.

Glucagon levels were affected by abdominal vagus nerve stimulation. When circulating levels of glucose in the blood stream become low, glucagon is released by α‐cells of the islet of Langerhans in the pancreas which stimulates the release of glucose in the liver in a process known as gluconeogenesis (Miller et al., 2013). Applying 15 Hz vagus nerve stimulation and afferent stimulation resulted in a substantial increase in glucagon levels. This finding is in line with the theory that electrically increasing the activity of afferent vagal fibers simulates an ‘artificial’ hypoglycemic state and causes the increased release of glucagon, and subsequently leads to hyperglycemia (Niijima, 1982, 1983).

Concurrently, inhibiting vagal (i.e., afferent) activity by applying 40 kHz stimulation leads to a substantial decrease in glucagon secretions. This is an important finding and may have application in the treatment of type 2 diabetes. Biguanides (brands include: Metformin, Diabex and Diaformin) are the most common class of medication used to control hyperglycemia of type 2 diabetes, and work by inhibiting glucose release from the liver by suppressing hepatic glucagon signaling (Miller et al., 2013). As such, glucagon‐lowering effects of the 40 kHz blocking stimulation strategy may potentially have useful application to the treatment of type 2 diabetes.

Previous studies demonstrate an increase in insulin levels (Table 1) in response to hyperglycemic levels when VNS is applied (Ahren et al., 1986; Ionescu et al., 1983). In this study no statistically significant changes in insulin were seen during the application of any electrical stimulation strategy, despite the substantial hyperglycemic effect seen during the application of VNS, aVNS, and 4 kHz stimulation strategies. However, significant hyperglycemia in the absence of insulin release during low‐frequency vagus nerve stimulation has been reportedly previously (Meyers et al., 2016), and is thought to be due to afferent‐dependent suppression of insulin release that is centrally mediated by the indirect activation of sympathetic splanchnic nerve, known to cause suppression of insulin release (Andersson et al., 1982; Dunning, Ahren, Veith, & Taborsky, 1988; Holst, Schwartz, Knuhtsen, Jensen, & Nielsen, 1986; Meyers et al., 2016).

GLP‐1 is another hormone that regulates glucose metabolism by promoting the release of insulin during post‐prandial phases of ingestion, and is a therapeutic target of type 2 diabetes medications. The vagus nerve extends branches into the gastrointestinal tract to innervate K‐ and L‐cells which produce the ‘incretin’ hormone GLP‐1 following ingestion of glucose (Berthoud, 2008). GLP‐1 promotes the release of insulin during postprandial phases of ingestion, and is a therapeutic target of a class of type 2 diabetic medications (GLP‐1 agonists). Although a previous study showed that low‐frequency electrical stimulation (20 Hz, 10 V) of the celiac vagal branch increased secretions of GLP‐1 (Rocca & Brubaker, 1999), in the current study we found inconsistent changes in the secretion of GLP‐1 during the application of different stimulation strategies. The difference between studies might be explained by the fact that the posterior celiac vagus nerve supplies the small and large intestine, whereas the nerve terminals of the anterior celiac nerve only supply as far as the duodenum (in rat) (Berthoud et al., 1991). However, in this study the left or anterior abdominal vagus nerve was stimulated (15 Hz). As such, stimulation of both the anterior and posterior vagus nerves could lead to an increase in GLP‐1 levels and provide potential therapeutic effect of decreasing glycemia.

A previous study achieved selective activation of cervical vagal afferent or efferent fibers by stimulating the cut proximal or distal end of the vagus nerve (Meyers et al., 2016). In this study, we achieved reversible directional stimulation by combining kilohertz‐frequency blocking with low‐frequency activation (Patel et al., 2017), and applied it to an abdominal location of the vagus nerve, close to the end organ. This is the first study that applied directional stimulation in order to control glycemia and hormone levels, and our technology allows for the long‐term assessment of this stimulation strategy in awake rats. Future studies should consider the effects of these stimulation strategies in a rat model of type 2 diabetes.

Inhalant isoflurane anesthesia was used in this study to enable long experimental testing to be conducted to evaluate the effects of different stimulation strategies. Isoflurane reportedly inhibits the release of insulin from the pancreas and impairs glucose tolerance and clearance in humans (Diltoer & Camu, 1988; Tanaka, Nabatame, & Tanifuji, 2005). Our results are consistent with a previous study that shows no increases in insulin secretions during elevated glucose levels induced by VNS in isoflurane‐anesthetized rats (Meyers et al., 2016). Furthermore, the suppression of insulin has been reported during application of VNS in awake rats, which resulted in elevated fasted blood glucose levels and an impaired glucose tolerance (Stauss et al., 2018). However, to remove the potential confounding effects of isoflurane, future studies should consider assessing the effect of various VNS stimulation strategies in awake animals.

In conclusion, electrical stimulation of the abdominal vagus nerve modulates glycemia by effecting glucagon secretions. Application of high‐frequency 40 kHz stimulation, which lowered glucagon levels, may have a potential application for the treatment of type 2 diabetes.

## ETHICS STATEMENT

Procedures were approved by the Bionics Institute Animal Research Ethics Committee (17‐369AB, 18‐384AB) and complied with the Australian Code for the Care and Use of Animals for Scientific Purposes (National Health and Medical Research Council of Australia) and the Prevention of Cruelty to Animals (1986) Act.

## CONFLICT OF INTEREST

All authors declare no conflicts of interest, financial or otherwise.

## AUTHOR CONTRIBUTIONS

All listed authors made substantial, direct and intellectual contributions to the study and manuscript.
